# Drivers of rabies post-exposure prophylaxis noncompletion in Cambodia, 2019 to 2022

**DOI:** 10.1371/journal.pntd.0013813

**Published:** 2025-12-18

**Authors:** Herman Banza Kongolo, Yiksing Peng, Malen Chan, Heidi Auerswald, Veasna Duong, Pisey Thai, Vechana Thap, Yin Hann, Aima Mohhamad, Florian Girond, Sowath Ly, Claude Flamand, Hélène Guis

**Affiliations:** 1 Epidemiology and Public Health Unit, Institut Pasteur du Cambodge, Phnom Penh, Cambodia; 2 ASTRE, Institut Pasteur du Cambodge, Phnom Penh, Cambodia; 3 ASTRE, University of Montpellier, CIRAD, INRAE, Montpellier, France; 4 Paris Cité University (Doctoral School 393 Pierre-Louis de Santé Publique), Paris, France; 5 Vaccination Service Unit, Institut Pasteur du Cambodge, Phnom Penh, Cambodia; 6 Virology Unit, Institut Pasteur du Cambodge, Phnom Penh, Cambodia; 7 Istituto Zooprofilattico Sperimentale dell’Abruzzo e del Molise (IZSAM) Teramo Campo Boario, Teramo, Italy; 8 Battambang, Rabies Prevention Center, Institut Pasteur du Cambodge, Phnom Penh, Cambodia; 9 Kampong Cham, Rabies Prevention Center, Institut Pasteur du Cambodge, Phnom Penh, Cambodia; 10 Communicable Disease Control Department, Ministry of Health, Phnom Penh, Cambodia; 11 Mathematical Modelling of Infectious Diseases Unit, Institut Pasteur, Université Paris Cité, U1332 INSERM, UMR2000 CNRS, Paris, France; Universidad Nacional Mayor de San Marcos, PERU

## Abstract

**Background:**

Achieving the “Zero by 30” goal—zero human deaths due to dog-mediated rabies by 2030—depends heavily on the timely and complete administration of post-exposure prophylaxis vaccination (PEPV) to exposed individuals. This study aims to assess trends and evaluate rabies PEPV noncompletion following the introduction of the abridged 1-week protocol, and identify its drivers in Cambodia, which bears a high rabies burden.

**Methodology:**

A retrospective analysis of patients attending the three Institut Pasteur du Cambodge (IPC) rabies prevention centers (RPCs) between 2019 and 2022 was conducted to assess exposure categories and PEPV completion. Multivariate regression was used to identify factors associated with noncompletion.

**Results:**

Among the 239,874 patients with a category II or III exposure requiring PEPV who attended the three IPC RPCs from 2019 to 2022, 6.4% did not complete the full three-session PEPV regimen or the two-session regimen for the previously immunized patients. Greater travel time to the RPC was strongly associated with lower attendance and completion rates. In addition, noncompletion was more common among males and working-age youth (15–29). Attendance and completion dropped sharply during Covid-19 mobility restriction periods and, conversely, rose markedly following a social media event publicizing a rabies-related death in 2019. Other factors significantly associated with PEPV noncompletion included year of PEPV administration, RPC location, species of the biting animal, health and living status, mode of attack and type of attack, and time between exposure and the first PEPV dose received.

**Conclusions:**

Although overall PEPV noncompletion rates were low at IPC’s RPCs, this study revealed significant temporal and spatial variations. Travel time to RPC emerged as a major barrier to both RPC attendance and PEPV completion, underscoring the need to decentralize PEPV delivery to reduce access inequities. High-risk groups, particularly males and youth (15–29), would benefit from targeted communication strategies and flexible service hours. These findings can inform rabies prevention strategies in Cambodia by guiding the development of more equitable and effective PEPV delivery systems—critical to achieve the “Zero by 30” goal.

## Author summary

Rabies is a neglected tropical disease that is nearly always fatal but entirely preventable through timely post-exposure prophylaxis vaccination (PEPV). In Cambodia, where rabies remains endemic, we analyzed PEPV completion among more than 240,000 patients who attended one of the three Institut Pasteur du Cambodge (IPC) Rabies Prevention Centers (RPCs) between 2019 and 2022. Overall, 6.4% of patients did not complete the full PEPV regimen. Multivariate analysis identified several factors significantly associated with noncompletion, including greater travel time to reach an RPC, socio-demographic factors (such as being aged 15–29, male gender), factors linked to the animal causing the exposure (such as dogs, and provoked attacks) and factors linked to PEPV itself (delays in initiating PEPV and not receiving rabies immunoglobulin (RIG)). Attendance and completion were also greatly impacted by Covid-19 mobility restrictions and a social media event publicizing a human rabies case leading to death in 2019. These findings highlight key barriers that hinder at-risk populations from completing lifesaving rabies PEPV. Improving geographic access to PEPV, enhancing targeted health education - particularly for younger working-age populations - and strengthening public awareness are essential to increasing completion rates. Addressing these gaps is essential for achieving the ‘Zero by 30’ goal of eliminating dog-mediated human rabies deaths by 2030.

## Introduction

Rabies is a zoonotic encephalitis caused by a lyssavirus transmitted to humans mainly by dogs, nearly always fatal after the onset of symptoms [1]. The estimated annual global death toll from rabies is approximately 59,000 humans [2], with children under 15 years old representing 40% of victims [2–4] and poor rural populations from low-income countries, especially in Asia and Africa, bearing the highest burden [2].

In 2018, the World Health Organization (WHO), the Food and Agriculture Organization of the United Nations (FAO), the World Organization for Animal Health (WOAH) and the Global Alliance for Rabies Control (GARC) have set a global goal of eliminating human deaths from dog-mediated rabies by 2030, known as the ’Zero by 30’ initiative [4]. To achieve this goal, three key strategies have been identified: (i) timely post-exposure prophylaxis (PEP) for exposed individuals [5–7], (ii) mass vaccinating of dogs to control the primary source of transmission to humans [8,9], and (iii) increased community awareness about rabies prevention [7,10].

Rabies PEP consists of immediate (i) wound washing with water and soap, (ii) vaccination against rabies virus (RABV) according to WHO-recommended protocols (thereafter called PEP vaccination, PEPV), and (iii) in case of severe exposure, administration of rabies immunoglobulin (RIG) in and around the wound(s) [3,11,12]. Patients should be informed of the critical need to initiate PEP without delay and to complete the full regimen of PEP administration to ensure effective protection and prevent death [3,6,12].

The development of the first rabies vaccine by Louis Pasteur in 1885 [13] paved the way to the production of safer and more immunogenic vaccines. Rabies vaccine formulations steadily improved over time, allowing progressively shorter intramuscular (IM) vaccination schedules. A substantial improvement to PEPV came in 2018 when WHO recommended an abridged 1-week intradermal (ID) regimen for rabies PEPV [3,14–16] after it had been shown to be as efficient as the 28-day ID four-session modified Thai Red Cross regimen [14,15], and as the 2-week IM four-session Essen regimen [17]. This dose-sparing, abridged regimen called Institut Pasteur du Cambodge regimen [12] involves two-site ID injections of 0.1 ml of vaccine at each site, administered on days 0, 3 and 7 [3,14–16]. However, timely access and completion with PEPV remain limited in many endemic countries, particularly among the most disadvantaged populations. Barriers include limited geographical access to facilities providing PEPV/RIG, as well as direct vaccine costs and indirect costs such as travel and lost income [18–22]. The Covid‑19 pandemic further strained access to care as disruptions to health services and fear of exposure to Severe Acute Respiratory Syndrome Coronavirus 2 (SARS-CoV-2) virus reduced health-seeking behaviors and ultimately led to a resurgence of rabies deaths [23–27].

In Cambodia, the estimated burden of rabies in 2007 was 810 human deaths (95% confidence interval [CI] 394–1,607), corresponding to an annual incidence of 5.8/100,000 (95% CI 2.8–11.5) [28], ranking among the highest in Asia [2]. The significant public health challenges posed by rabies in Cambodia prompted the Institut Pasteur du Cambodge (IPC) to establish a first Rabies Prevention Center (RPC) in Phnom Penh in 1995, following a request from local authorities. To meet the growing demand for PEP and expand access to treatment, two additional centers were later opened in Battambang (2018) and Kampong Cham (2019). From 2009 to 2013, an overall 7.8% noncompletion rate was observed among patients receiving PEPV at the RPC in Phnom Penh [29]. At that time, the recommended PEPV protocols consisted of five and, later, four sessions of ID vaccine doses over 90 and 28 days, respectively [29]. The study showed that noncompletion increased with the length of PEPV regimens, distance of patients to RPC, in males, the younger working-age group (15–29 years), when the dog was considered healthy by the victim and when the initial visit occurred during the rice harvest season corresponding to work commitments [29]. These factors are concordant with other studies on rabies PEPV completion [18,20,30–34]. However, some results were unexpected: noncompletion rates increased over the years, and were higher when the attack was unprovoked or when the dog had disappeared [29].

This study analyzes data from all three IPC RPCs from 2019 to 2022 to assess PEPV completion rates and identify their determinants within a rapidly evolving context. During the study period, the abridged 1-week PEPV protocol was already in use, having been introduced in June 2018, and two additional RPCs had recently been established June 2018 and March 2019. Two contextual events likely to influence PEPV-seeking behaviors and adherence occurred: (i) the Covid-19 pandemic, which disrupted health service access and movement and (ii) a widely publicized case in February 2019 involving the death of a young girl after suspected rabies exposure from a cat [35]. The case, relayed through a viral social media appeal from the child’s mother, received national attention and triggered a surge in attendance at the Phnom Penh RPC.

This study aims to assess trends in PEPV completion following the introduction of the abridged 1-week protocol, and to compare completion rates across the three IPC RPCs. It further evaluates the impact of external events, including Covid-19 restrictions and a widely circulated rabies-related social media content in 2019, on PEPV adherence. In addition, the study explores spatial and temporal variations in completion and identifies individual and contextual factors associated with PEPV noncompletion. By addressing these objectives, the study aims to inform more effective rabies prevention strategies and contribute to Cambodia’s progress toward achieving the ‘Zero by 30’ goal.

## Materials and methods

### Study design and data collection

This retrospective study used data routinely collected at the three IPC RPCs, located in Phnom Penh, Battambang and Kampong Cham. Upon arrival, all patients attending the RPCs completed a standardized paper-based questionnaire. The questionnaire was designed to collect: (i) socio-demographic information (e.g., age, sex, address), (ii) details of the exposure event, (iii) information about the animal involved, (iv) actions taken by the patient following exposure, and (v) data on the timing and number of PEPV doses received as well as the use of RIG and previous rabies immunization (S1 File). Data on dog or cat vaccination status against rabies virus were not collected. Overall dog vaccination coverage in Cambodia is extremely low. Questionnaire data were entered and stored in a RedCap database (version 12.5.5).

### Inclusion and exclusion criteria

**Fig 1:**
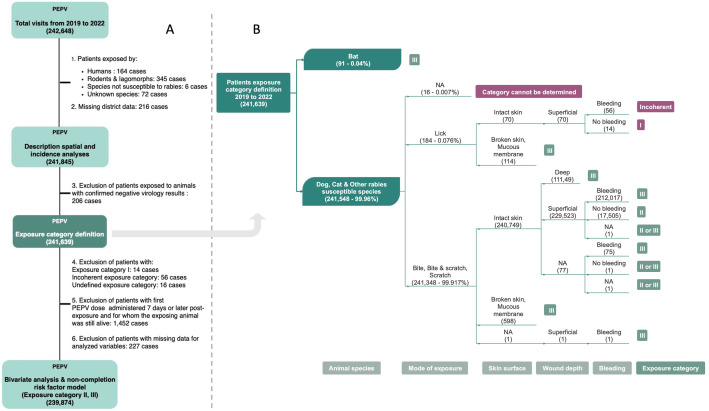
Flow diagram of patient inclusion and exposure category assignment for PEPV noncompletion analysis, 2019-2022. (A) Stepwise inclusion of patients from the initial cohort of 242,648 individuals who attended the three IPC Rabies Prevention Centers (RPCs) between 2019 and 2022. Exclusions were applied based on species not known to transmit rabies (humans, rodents, lagomorphs, etc.), missing district data, confirmed negative virology results in the exposing animal, Category I or undefined exposures, late PEP initiation with the animal still alive at day 10 post-exposure, and missing data for key variables, resulting in a final analytic cohort of 239,874 patients. (B) Classification of patients (n = 241,639) based on WHO exposure categories using information on the animal species, mode of exposure (bite, scratch, lick), skin surface (intact or broken/mucosal), wound depth (superficial or deep), and presence of bleeding. Exposure categories were assigned as Category II, III, or mixed (II or III); a small number of cases were classified as “incoherent” due to inconsistent data or as “category cannot be determined” to indicate cases where exposure type was missing and could not be classified into a WHO exposure categories. Color-coded boxes represent categorical decisions, while icon labels indicate the exposure classification pathway based on animal species, exposure tyspe, and clinical characteristics..

Rabies exposure was categorized following current WHO guidelines [12]. Category I exposures (touching or feeding animals, or licks on intact skin) do not require PEPV. In contrast, category II exposures (nibbling of uncovered skin, minor scratches or abrasions without bleeding) and category III exposures (single or multiple transdermal bites or scratches, saliva contact with mucous membranes or broken skin, and direct contact with bats) necessitate PEPV. RIG is recommended in addition to PEPV for category III exposures, but in contexts where a limited amount of RIG is available, WHO recommends to prioritize it to high-risk exposures, which include: multiple bites; deep wounds; bites to highly innervated areas such as the head, neck and hands; severe immunodeficiency; bites from animals with laboratory confirmed or suspected rabies; and bites, scratches or mucous membrane exposures resulting from contact with bats [16]. As the exposure category was not recorded directly, it was retrospectively classified based on the animal species, exposure mode (bite, scratch, lick, or combination), contact surface (intact/broken skin, mucosa), wound severity (superficial or deep), and presence of bleeding (yes or no) (Fig 1B).

For descriptive, spatial and incidence analyses, we included patients exposed to rabies-susceptible mammals (dogs, cats, monkeys, cows etc.) between 2019 and 2022. Patients exposed to humans, rodents, lagomorphs, non-transmitting species or unidentified species were excluded. Spatial analyses were performed at the district level; patients with missing or invalid district information were excluded.

For the risk factor analysis of PEPV noncompletion, we further excluded: (i) patients exposed to animals that tested negative for RABV (ii) patients with category I exposures or for whom the exposure category could not be determined, (iii) patients whose first PEPV dose was administered seven days or later after exposure, in cases where the animal remained alive at day 10 post-exposure, and (iv) patients with missing data (NA) for key variables. Patients exposed to animals that tested negative for RABV were excluded because they do not require PEPV and typically discontinue vaccination upon receipt of virological results of the animal [12]. Similarly, patients who began PEPV on or after day 7 following exposure and whose biting animal remained healthy 10 days after the exposure can also interrupt PEPV as these cases indicate that the animal was not rabid at the time of the incident.

### RPC PEPV and completion definition

During the study period, all three IPC RPCs administered PEPV using the VERORAB vaccine (Sanofi, Lyon, France), following a three-session ID protocol consisting of two-site injections on days 0, 3, and 7. Patients with no prior history of rabies vaccination were considered complete if they completed all three sessions. Individuals with a documented history of full prior PEPV were classified as complete if they received two sessions following a new exposure. Patients who missed one or more required sessions were classified as incomplete. If a scheduled session fell on a Sunday, when the RPCs were closed, patients were advised to receive the dose either one day earlier or later; therefore, exact adherence to the day-specific schedule was not considered in the completion assessment. Patients who delayed or temporarily interrupted the schedule were still considered complete, provided they completed the required number of sessions.

It is to be noted that a partial cost recovery scheme is in place in the IPC RPCs. Patients who receive PEPV at IPC RPCs are asked to pay US$15fee for the full three-session PEPV course on the day of the first PEPV session. This fee is lower than the wholesale cost (the cost for the practitioner purchasing the vaccine excluding the additional costs of transport, storage, injection material, personnel and infrastructure), IPC subsidizes the difference and the additional costs. Due to the limitations in tracing all severe cases via the questionnaire, RIG administration was not included in the assessment of PEPV completion. For patients unable to afford treatment, IPC subsidizes entirely PEPV (and RIG if needed) so as to leave no exposed patient untreated.

### Data analysis

Each exposed patient visiting an RPC was classified as either complete or incomplete with the PEPV protocol. We calculated the incidence of noncompletion and mapped it using district-level population data from the 2019 general population census [36].

We assessed bivariate statistical associations between PEPV noncompletion and 17 explanatory variables, including 15 questionnaire-derived variables (covering demographic, exposure-related, behavioral characteristics and travel time from patients’ district centroid to the visited RPC) and two contextual variables reflecting potential external influences on PEPV completion: a widely publicized social media event in February 2019 and the national Covid-19 restrictions implemented from 2020 to 2021. All questionnaire-derived variables were categorical, except patient age and the travel time between the centroid of the patient’s district of residence and the RPC which were initially continuous but re-coded into categorical variables for analysis.

The Travel times between Cambodian districts and RPCs were computed using a cost-distance modeling approach, with district centroids defined as destination points, while centroids of districts hosting RPCs served as origins. The 2019 motorized friction surface from the Malaria Atlas Project was used as the base layer [37], with pixel value representing the cost (minutes per meter) required to cross different terrains and road networks. The friction surface was clipped to Cambodia’s administrative boundaries (GADM level 0) [38]. A transition matrix was created by inverting friction values to derive conductance (travel speed) and corrected geometrically before calculating cumulative travel costs with terra [39] and gdistance package [40]. The resulting matrix provided estimated travel times (in minutes) from each district to the three RPCs. Districts were then assigned to their nearest center based on minimum travel time, enabling population-weighted accessibility analyses. For districts with missing travel time estimation, we applied the travel time of the district with the closest centroid. To better reflect the effective accessibility experienced by the overall population, we computed the population-weighted median travel time, assigning each district a weight proportional to its 2019 population size [36].

The social media event was defined as the three-month period from February to April 2019, during which a widely circulated public appeal described the death of a child from suspected rabies following cat exposure. This event triggered a notable surge in attendance at the Phnom Penh RPC [35]. To assess its impact, we created a binary time variable indicating whether a patient’s visit occurred during this event window or at any other time.

The Covid-19 restriction period variable was defined based on government-implemented public health measures, including lockdowns, school closures, and bans on public gatherings, after the first confirmed Covid-19 case in Cambodia on January 27^th^, 2020 [41]. We classified the following months as “Covid-19 restriction months”: March–April 2020 (initial national restrictions), November–December 2020 (response to a large community cluster), and April–May 2021 (nationwide lockdown). A binary indicator was used to distinguish these months from the rest of the study period.

All 17 explanatory variables were tested for bivariate associations with the binary outcome of PEPV noncompletion using Pearson’s Chi-square test. Variables with a p-value <0.05 were considered statistically significant and included in a multivariate logistic regression model to estimate odds ratios (ORs) with Wald 95% confidence intervals. Model selection was based on the lowest Akaike Information Criterion (AIC), and model performance was assessed by calculating the area under the receiver operating characteristic curve (AUC-ROC). Statistical and spatial analyses were performed using R software [42].

## Results

### Study population

Between 2019 and 2022, a total of 242,648 patients attended the three IPC RPCs. After excluding patients exposed to species not susceptible to rabies (6 cases), humans (164), rodents and lagomorphs (345), unknown species (72), and those with missing district data (216), 241,845 patients (99.7%) were included in the descriptive, spatial, and incidence analyses. The Phnom Penh RPC accounted for 61.6% of visits (149,080 patients), Battambang RPC with 23.3% (56,441), and Kampong Cham RPC with 15% (36,324) (Table 1).

**Table 1:** Annual number of patients seeking post-exposure prophylaxis vaccination (PEPV) and number of patients with incomplete PEPV, by year and by Rabies Prevention Center (RPC).

Year	Number of patients seeking PEPV	Rabies Prevention Center			Total per year
		Phnom Penh	Battambang	Kampong Cham	
2019	Total (%)	52,237 (67.1%)	14,903 (19.1%)	10,756 (13.8%)	77,896 (32.2%)
	with incomplete PEPV (%)	3,362 (6.4%)	527 (3.5%)	7 (<0.1%)	3,896 (5%)
2020	Total (%)	34,499 (60.5%)	12,899 (22.6%)	9,590 (16.8%)	56,988 (23.6%)
	with incomplete PEPV (%)	2,847 (8.3%)	755 (5.9%)	499 (5.2%)	4,101 (7.2%)
2021	Total (%)	25,232 (57.2%)	12,207 (27.7%)	6,645 (15.1%)	44,084 (18.2%)
	with incomplete PEPV (%)	1,651 (6.5%)	782 (6.4%)	222 (3.3%)	2,655 (6%)
2022	Total (%)	37,112 (59%)	16,432 (26.1%)	9,333 (14.8%)	62,877 (26%)
	with incomplete PEPV (%)	3,205 (8.6%)	1,054 (6.4%)	532 (5.7%)	4,791 (7.6%)
**Total per center**	Total (%)	149,080 (61.6%)	56,441 (23.3%)	36,324 (15%)	241,845 (100.0%)
	with incomplete PEPV (%)	11,065 (7.4%)	3,118 (5.5%)	1,260 (3.5%)	15,443 (6.4%)

Exposure categories, defined according to WHO guidelines, were assigned for 241,639 patients (99.6%) (Fig 1B), after the exclusion of 206 patients due to the negative result from the RABV laboratory test. Among these, 224,045 (92.7%) were classified as category III exposures, 17,505 (7.2%) as category II, 3 patients had mixed category II/III exposures due to lack of information on wound bleeding, and 14 were classified as category I. Exposure category could not be defined for 16 patients and was considered incoherent for 56 patients (Fig 1B).

For the analysis of factors associated with PEPV completion, we further excluded from the 241,639 patients from whom the exposure category was defined: patients with category I exposure (14 patients), with an incoherent exposure category (56 patients), for whom the exposure category could not be defined (16 patients), patients who initiated PEPV on or after day 7 post-exposure while the animal remained alive (1,452 patients), and those with missing key variables (227 patients) (Fig 1A). We therefore focused on 239,874 patients (representing 98.9% of all attendees) with an exposure category II or III (S1 Fig). All 239,874 patients initiated PEPV, and 93.6% completed the full three-session regimen for naive patients or two-session regimen for those previously immunized (S2 Fig).

### Temporal variations

Over the four-year study period, the mean annual attendance at the three RPCs was 60,461 patients (±14,024 standard deviation (SD)). Attendance was lowest in 2021, with 44,084 patients (18.2%), and highest in 2019, with 77,896 patients (32.2%), particularly in Phnom Penh and Kampong Cham (Table 1). In contrast, Battambang RPC reached its peak attendance in 2022. The incidence of exposure per 100,000 inhabitants mirrored this trend, peaking in 2022 at 400.9.

Overall, 6.4% of patients did not complete the PEP regimen. Among these incomplete patients, the majority had initiated PEPV promptly 66.7% (10,231) within 1 day, 25.8% (3,967) within 2–3 days, and 7.5% (1,151) within 4–6 days post-exposure. The rate of noncompletion increased significantly from 5.0% in 2019 to 7.6% in 2022 (p < 0.001; Table 2). Notably, noncompletion rates were significantly lower during the 2019 social media event (3.2%) compared to other periods (6.8, p < 0.001). In contrast, rates were higher during periods of Covid-19 restrictions (7.3) compared to non-restriction periods (6.3%, p < 0.001; Table 2).

**Table 2:** Socio-demographic characteristics of PEPV patients and noncompletion rates.

Characteristic	Overall, N = 239,874	Complete, N = 224,525	Incomplete, N = 15,349	p-value^a^
Year, n (%)				<0.001
-*2019*	77,069 (32.1%)	73,224 (95.0%)	3,845 (5.0%)	
-*2020*	56,650 (23.6%)	52,560 (92.8%)	4,090 (7.2%)	
-*2021*	43,779 (18.3%)	41,133 (94.0%)	2,646 (6.0%)	
-*2022*	62,376 (26.0%)	57,608 (92.4%)	4,768 (7.6%)	
**Rabies Prevention Center, n (%)**				<0.001
-*Battambang*	56,166 (23.4%)	53,085 (94.5%)	3,081 (5.5%)	
-*Kampong Cham*	36,089 (15.0%)	34,832 (96.5%)	1,257 (3.5%)	
-*Phnom Penh*	147,619 (61.5%)	136,608 (92.5%)	11,011 (7.5%)	
**Travel time (Hour), n (%)**				<0.001
- ≤ 1 Hour	171,285.0 (71.4%)	160,468.0 (93.7%)	10,817.0 (6.3%)	
-1–2 Hours	48,704 (20.3%)	45,800 (94.0%)	2,904 (6.0%)	
-2–3 Hours	13,910 (5.8%)	12,907 (92.8%)	1,003 (7.2%)	
-3–4 Hours	2,778 (1.2%)	2,515 (90.5%)	263 (9.5%)	
- ≥ 4 Hours	3,197 (1.3%)	2,835 (88.7%)	362 (11.3%)	
**Age group, n (%)**				<0.001
-* < 15 years*	108,667 (45.3%)	102,967 (94.8%)	5,700 (5.2%)	
-*15–29 years*	43,765 (18.2%)	39,606 (90.5%)	4,159 (9.5%)	
-*30–44 years*	43,193 (18.0%)	40,051 (92.7%)	3,142 (7.3%)	
-*45–59 years*	27,305 (11.4%)	25,806 (94.5%)	1,499 (5.5%)	
-* ≥ 60 years*	16,944 (7.1%)	16,095 (95.0%)	849 (5.0%)	
**Gender, n (%)**				<0.001
-*Female*	123,290 (51.4%)	116,034 (94.1%)	7,256 (5.9%)	
-*Male*	116,584 (48.6%)	108,491 (93.1%)	8,093 (6.9%)	
**Social media event, n (%)**				<0.001
-*Before & after event*	215,643 (89.9%)	201,060 (93.2%)	14,583 (6.8%)	
-*During event*	24,231 (10.1%)	23,465 (96.8%)	766 (3.2%)	
**Covid-19 restriction period, n (%)**				<0.001
-*Non-restriction months*	215,574 (89.9%)	202,005 (93.7%)	13,569 (6.3%)	
-*Restriction months*	24,300 (10.1%)	22,520 (92.7%)	1,780 (7.3%)	

^a^p values were calculated using Chi-square test.

**Fig 2:**
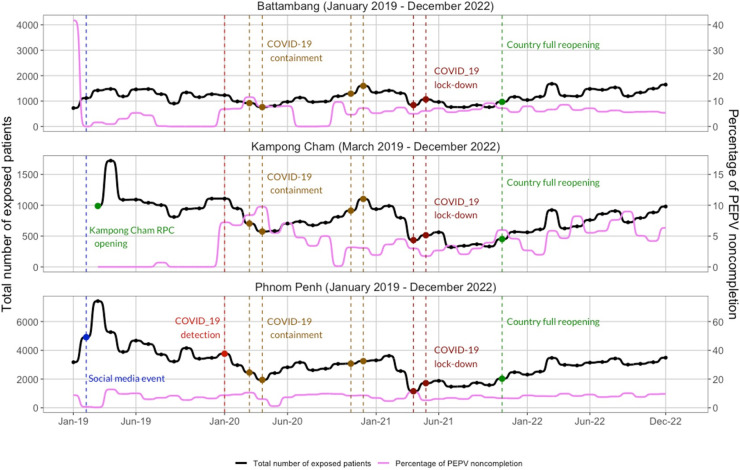
Temporal trends in patient attendance and PEPV noncompletion across the three IPC Rabies Prevention Centers (RPCs), 2019-2022. Monthly trends show the total number of patients with WHO-defined Category II or III exposures requiring PEPV (black line, left y-axis) and the corresponding percentage of patients who did not complete the full PEPV regimen (pink line, right y-axis) at each RPC: Battambang, Kampong Cham, and Phnom Penh. Key contextual events are annotated, including the opening of the Kampong Cham RPC, a high-profile social media event (Feb–Apr 2019), Covid-19 detection and containment phases, nationwide lockdowns, and the full reopening of the country. Data include all patients eligible for PEPV from 2019 to 2022 (n = 239,874).

Monthly analyses showed a sharp increase in the number of exposed patients from February to March 2019, especially in Phnom Penh and Kampong Cham RPCs, accompanied by near-zero noncompletion rates (Fig 2). In Kampong Cham, high completion rates persisted throughout 2019 In contrast, Battambang experienced only moderate increases in patient volume during this period but showed elevated noncompletion rates between January and July 2019.

Patient attendance decreased between February and May 2020, coinciding with the onset of Covid-19 restrictions such as school closures and bans on public gatherings (Fig 2). A similar drop occurred from March to May 2021, especially in Phnom Penh. Despite these challenges, Kampong Cham RPC consistently maintained high completion rates, in contrast to Phnom Penh and Battambang, where noncompletion rates were significantly higher (p < 0.001, Table 2). Following the nationwide reopening in November 2021, attendance steadily increased across all three RPCs (Fig 2).

### Accessibility to Institut Pasteur du Cambodge rabies prevention centers

Travel times were estimated for 591 origin–destination pairs, representing accessibility from 197 districts to the three IPC RPCs. Travel time could not be calculated for the district of Preah Sihanouk (Preah Sihanouk province) because the district includes both mainland and island areas, placing its centroid between them. Therefore, it was assigned the travel time of the nearest district centroid, that of Stueng Hav district (also in Preah Sihanouk province). Preah Sihanouk district accounted for 105 patients (0.04%).

S3 Fig shows the travel time from each district to Battambang (S3A Fig), Kampong Cham (S3B Fig) and Phnom Penh (S3C Fig) RPCs. The median travel time from all districts to Battambang RPC was 413.99 minutes (6.9 hours) (297.92 – 506.08 interquartile range (IQR)) and the maximum travel time (from districts in the northern provinces of Stoeng Treng and Ratanakiri; the eastern provinces of Kratie, Mondolkiri, Tboung Khmum, Prey Veng and Svay Rieng; and the southern provinces of Takeo, Kampot and Preah Sihanouk) was 1109.04 minutes (18.5 hours) (S3A Fig). For the Phnom Penh RPC the overall median travel time was 216.57 minutes (3.6 hours) (106.1 – 424.57 IQR) and the maximum (from districts of Mondolkiri, Ratanakiri, Stoeng Treng, Oddar Meanchey and Banteay Meanchey provinces) was 863.18 minutes (14.4 hours) (S3B Fig). The Kampong Cham RPC showed an overall median accessibility of 232.56 minutes (3.9 hours) (136.58 – 411.9 IQR) and a maximum (from districts of Ratanakiri, Oddar Meanchey, Banteay Meanchey, Pailin and Koh Kong provinces) of 758.42 minutes (12.6 hours) (S3C Fig).

According to these theoretical travel times, Phnom Penh RPC was the most accessible with the shortest median travel time (3.6 hours), followed by Kampong Cham (3.9 hours) and Battambang (6.9 hours). However, Kampong Cham RPC had the shortest maximum travel time (12.6 hours), indicating that it was accessible from its most remote districts in less time than was required to reach Phnom Penh (14.4 hours) or Battambang (18.5 hours) from their respective outermost districts. This reflects Kampong Cham’s slightly more balanced geographical position and effective regional coverage across central and eastern provinces. In contrast, Battambang exhibited both the highest median and maximum travel times, highlighting persistent accessibility challenges, including from districts near the Tonlé Sap Lake.

**Fig 3:**
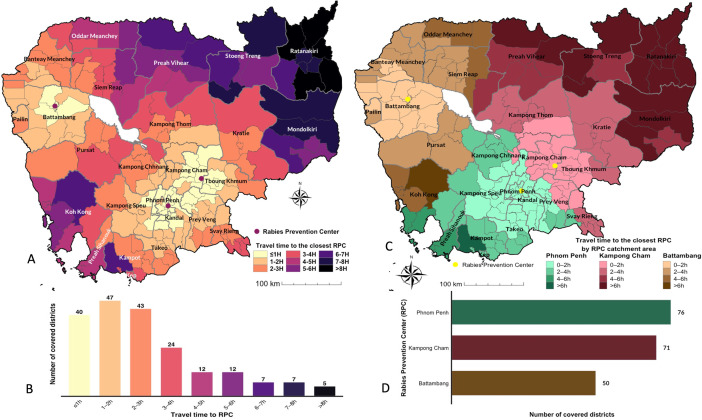
Travel time from districts to RPCs. (A) Map of travel time from districts to the RPC that is temporally the closest. (B) Number of districts by travel time. (C) Travel time to the closest RPC by RPC catchment area. (D) Number of districts temporally closest to each RPC. Travel time was computed using the terra and gdistance packages in R software, based on the Malaria Atlas Project, Global Motorized Friction Surface 2019. The Malaria Atlas Project maps are under the “Creative Commons Attribution 3.0 Unported License” (https://malariaatlas.org/open-access-policy/). The source of the basemap shapefile is https://gadm.org/download_country.html. The data are freely available for academic use such as publishing of academic research articles https://gadm.org/license.html.

Fig 3A illustrates the minimum travel time required for each district to reach the RPC that is temporally closest. It shows that districts located on a north-west to south-east diagonal have the shortest time travel to RPCs. It is worth noting that this diagonal corresponds to the areas of highest population density (S4 Fig). Inversely, with travel times exceeding three hours, the northern and north-eastern provinces of Mondolkiri, Ratanakiri, Stoeng Treng, Preah Vihear and Oddar Meanchey and Koh Kong, Preah Sihanouk and Kampot province in the south-west have the poorest accessibility to the three RPCs (Fig 3A). Most districts (66%), representing 80.2% of population, are accessible within 3 hours of one of these three RPCs (Fig 3B). Phnom Penh RPC is temporally closest to the highest number of districts (76), followed by Kampong Cham (71), then Battambang RPC (50) (Fig 3C). In terms of population theoretically covered by each RPC, Phnom Penh RPC encompasses 50.4% of the 2019 national population - 15,552,211 [36], Kampong Cham RPC 27.3% and Battambang RPC 22.3%. Thus, Phnom Penh provides the broadest interprovincial reach, reflecting its central geographic location and high road density (Fig 3D). Considering the spatial distribution of the population, the theoretical median travel time for all Cambodians to the RPC that was temporally closest (i.e., with the shortest travel time) was 91.18 minutes (42.15 – 162.63 IQR).

### Patient travel time and spatial inequities

Nearly all patients (91.7%, 219,989) visiting the RPCs came from districts located within 2 hours of access of an RPC: 71.4% (171,285) lived within one hour from an RPC and 20.3% (48,704) of patients lived between 1–2 hours from an RPC (Table 2). Only 5.8% came from a district located 2–3 hours away and 2.5% from districts located more than 3 hours away. PEPV noncompletion increased significantly with longer travel time: approximately 6.2% among patients residing within two hours of an RPC, 7.2% among those 2–3 hours away, 9.5% among those 3–4 hours away and 11.3% among those living more than 4 hours from RPC (p < 0.001; Table 2).

**Fig 4:**
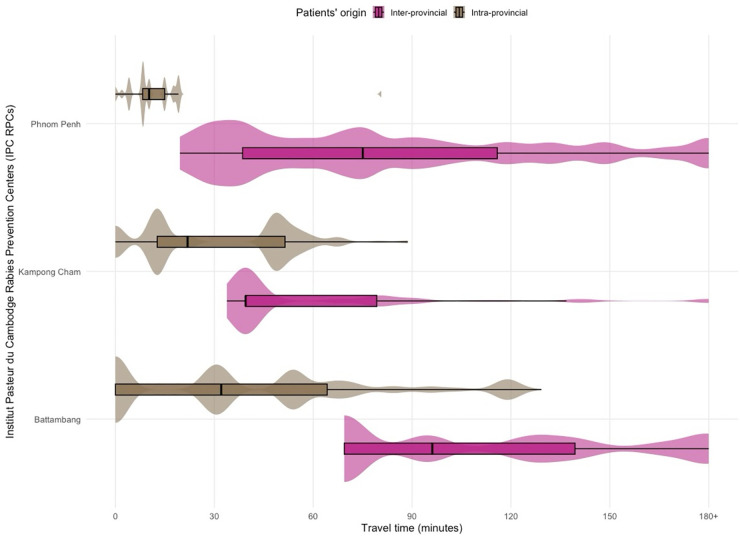
Patient travel time to the three RPCs from 2019 to 2022. The figure compares the variability of travel time between patients living within the same province hosting the RPC (intra-provincial, in brown) and those living outside (inter-provincial, in pink). For each RPC, the violin shows the density of patient travel time to the visited RPC, and the boxplot shows median and interquartile range (IQR) of patient travel time.

The patient’s overall median travel time was 32.1 minutes (10.2 – 68.0 IQR). It was 53.9 minutes (0 – 69.7 IQR) for those in Battambang, 39.5 minutes (12.7 – 51.4 IQR) for those in Kampong Cham and 19.6 minutes (8.4 – 75.4 IQR) for those in Phnom Penh, which has the largest number of patients (Fig 4). However, when focusing only on patients living in another province than the one hosting the visited RPC, the median accessibility time increased significantly to 96.2 minutes (69.4 – 139.4 IQR) for Battambang RPC and 75.1 minutes (38.6 – 115.9 IQR) for Phnom Penh RPC. Conversely, the median accessibility time did not change for Kampong Cham RPC, 39.5 minutes (39.5 – 79.3 IQR) (Fig 4).

Across all patients presenting with category II or III exposures, the proportion of individuals residing outside the province hosting the visited RPC was 10.6% for Battambang, 26.5% for Kampong Cham, and 49.0% for Phnom Penh.

The median patient travel time to the visited RPC differed significantly between Covid-19 restriction and non-restriction months (Wilcoxon rank-sum test, p < 0.001). Overall, travel time was shorter during restriction months (28.9 minutes, IQR: 10.2–59.8) than during non-restriction months (32.1 minutes, IQR: 10.2–69.4). This reduction was significant in Battambang and Phnom Penh RPCs (p < 0.001), with travel times decreasing from 53.9 minutes (IQR: 0.0–69.7) to 32.1 minutes (IQR: 0.0–69.7) in Battambang, and from 19.6 minutes (IQR: 10.2–75.5) to 19.1 minutes (IQR: 8.4–58.6) in Phnom Penh. No significant difference was observed in Kampong Cham (p = 0.08; 39.5 minutes, IQR: 12.7–51.4, for both periods).

### Spatial variations of patients’ visits

**Fig 5:**
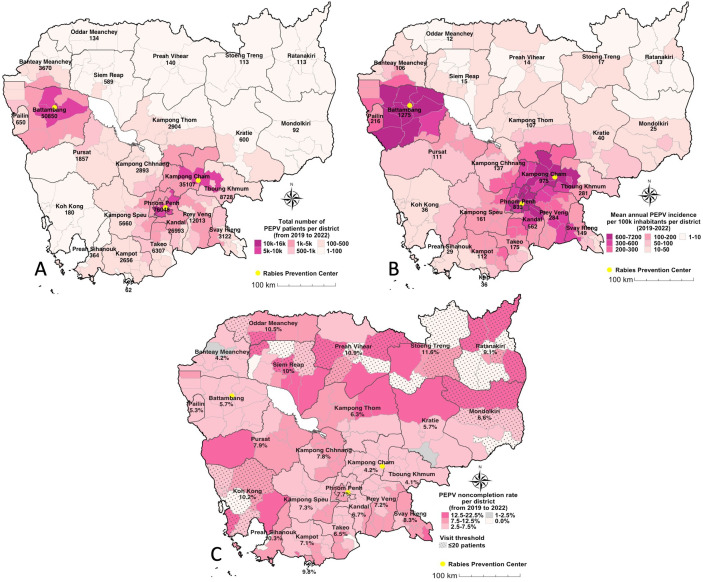
Spatial distribution of PEPV attendance, incidence, and noncompletion rates in Cambodia, 2019 to 2022. (A) Total number of patients seeking PEPV per province. (B) Mean annual incidence of PEPV patients per 100,000 inhabitants. (C) PEPV noncompletion rates by province, limited to districts with >20 patients. Panels A and B were based on the total number of patients included in the descriptive, spatial, and incidence analyses (n = 241,845), while Panel C includes only patients with WHO-defined category II or III exposures necessitating PEPV (n = 239,874)). The maps were created using R software. The source of the basemap shapefile is https://gadm.org/download_country.html. The data are freely available for academic use such as publishing of academic research articles https://gadm.org/license.html.

Provinces hosting RPCs had the highest incidence of patient visits per 100,000 population: Battambang (1,275 patients per 100,000 inhabitants), Kampong Cham (975), and Phnom Penh (833) (Fig 5A). The mean district-level incidence was 323.6 cases per 100,000 inhabitants (±685.7 SD), with a median of 105.8 (26.6 - 287.9 interquartile range (IQR)). An exceptionally high incidence was observed in districts with small populations (S4 Fig), such as Chamkar Leu (7,122 cases per 100,000) in Kampong Cham province. Ten remote provinces reported less than 50 exposures per 100,000 (Oddar Meanchey, Stoeng Treng, Siem Reap, Preah Vihear, Ratanakiri, Kratie, Mondolkiri, Koh Kong, Preah Sihanouk, and Kep).

Among districts with more than 20 patients, PEPV noncompletion rates ranged from 0.0% to 22.2%, with an average of 7.3% (± 3.3 SD; Fig 5C). Eight of the ten provinces with the lowest incidence also reported elevated noncompletion rates, notably Stoeng Treng (11.6%), Preah Vihear (10.9%), Oddar Meanchey (10.5%), Preah Sihanouk (10.3%), Koh Kong (10.2%), Siem Reap (10%), Kep (9.8%), and Ratanakiri (9.1%) (Fig 5C).

### Patient characteristics

**Fig 6:**
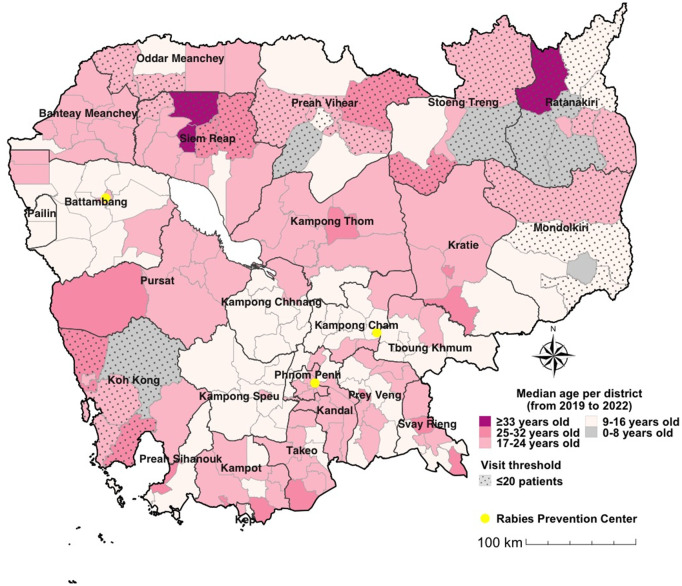
Spatial distribution of the median age of patients seeking PEPV, 2019-2022. The map shows the median age of patients by district across Cambodia, based on all included visits (n = 241,845). Shading indicates age categories, and districts with fewer than 20 patients are shown with a dotted pattern. Median ages ranged from 13 to 24 years, with younger median ages observed in districts near newly established RPCs. The map was created using R software. The source of the basemap shapefile is https://gadm.org/download_country.html. The data are freely available for academic use such as publishing of academic research articles https://gadm.org/license.html.

Patient ages ranged from 0 to 103 years, with a median age of 18 years (6–38 IQR) and a mean age of 24.2 years (±20.2 SD). At the provincial level, the median patient age varied from 13 years in Pailin to 24 years in Siem Reap. In most provinces (14 out of 25), the median patient age was between 15 and 18 years (Fig 6). Districts in or near the newly established RPCs - including Battambang, Pailin, Kampong Cham, Tboung Khmum, Kampong Chhnang, and Kampong Speu - had lower median patient ages, ranging from 9 to 16 years. In contrast, districts located farther from RPCs generally exhibited higher median patient ages (Fig 6). Nearly half (45.3%) of PEPV patients were under 15 years old, and 51.4% were female. PEPV noncompletion was significantly higher among patients aged 15–29 years (9.5%) and among males (6.9%) compared to their counterparts (p < 0.001; Table 2).

### Exposure characteristics

Dogs were the main source of exposure, accounting for 59.2% (142,093) of cases, followed by cats (39.9%, 95,760), while other species represented only 0.8% (2,021; Table 3). Most animals were owned (88.3%, 211,754) and reported as healthy at the time of exposure (97.6%, 234,126). The majority of exposures involved bites or bites with scratches (88.1%, 211,303), and over half were unprovoked (53.2%, 127,500).

**Table 3:** Characteristics of the animals involved in exposure events and their association with PEPV noncompletion.

Characteristic	Overall, N = 239,874	Complete N = 224,525	Incomplete N = 15,349	p-value
Animal species, n (%)				<0.001
-*Cat*	95,760 (39.9%)	90,009 (94.0%)	5,751 (6.0%)	
-*Dog*	142,093 (59.2%)	132,623 (93.3%)	9,470 (6.7%)	
-*Other species*	2,021 (0.8%)	1,893 (93.7%)	128 (6.3%)	
**Animal ownership, n (%)**				0.3
-*Owned*	211,754 (88.3%)	198,246 (93.6%)	13,508 (6.4%)	
-*Stray | Feral*	28,120 (11.7%)	26,279 (93.5%)	1,841 (6.5%)	
**Animal health status, n (%)**				<0.001
-*Healthy*	234,126 (97.6%)	218,980 (93.5%)	15,146 (6.5%)	
-*Sick*	5,748 (2.4%)	5,545 (96.5%)	203 (3.5%)	
**Exposure mode, n (%)**				<0.001
-*Lick/ Scratch*	28,571 (11.9%)	26,960 (94.4%)	1,611 (5.6%)	
-*Bite/ Bite & scratch*	211,303 (88.1%)	197,565 (93.5%)	13,738 (6.5%)	
**Attack type, n (%)**				0.018
-*Provoked*	112,374 (46.8%)	105,042 (93.5%)	7,332 (6.5%)	
-*Unprovoked*	127,500 (53.2%)	119,483 (93.7%)	8,017 (6.3%)	
**Animal living status, n (%)**				<0.001
-*Accessible*	210,521 (87.8%)	196,748 (93.5%)	13,773 (6.5%)	
-*Missing*	17,943 (7.5%)	17,014 (94.8%)	929 (5.2%)	
-*Spontaneous death*	2,054 (0.9%)	1,965 (95.7%)	89 (4.3%)	
-*Slaughtered*	9,275 (3.9%)	8,719 (94.0%)	556 (6.0%)	
-Slaughtered or Spontaneous death*	81 (0.0%)	79 (97.5%)	2 (2.5%)	
**Animal RABV laboratory result, n (%)**				<0.001
-*Not tested*	239,213 (99.7%)	223,883 (93.6%)	15,330 (6.4%)	
-*Rabid*	661 (0.3%)	642 (97.1%)	19 (2.9%)	

* Tested animals with ambiguous status on reason of death.

PEPV noncompletion was significantly higher following dog exposures (6.7%) and exposures to other species (6.3%) compared to cat exposures (6.0%, p < 0.001; Table 3). No significant difference in noncompletion was found between exposures from owned versus non-owned (stray or feral) animals (p = 0.3). However, noncompletion was higher when the animal was healthy at the time of exposure (6.5%) compared to sick (3.5%, p < 0.001). Exposures through licks were rare, accounting for 1,259 cases (0.21% of total exposures), of which 163 (0.03%) were classified as category III. Among patients included in PEPV noncompletion analysis, patients exposed through licks on broken skin or mucous membranes (category III according to WHO) were grouped with those exposed through scratches, totaling 28,571 cases (11.9% of all exposures). Among them, 1,611 patients (5.6%) did not complete their PEPV. PEPV noncompletion rate was higher for bites or bites with scratches (6.5%) compared to licks or scratches (5.6%, p < 0.001). Provoked exposures were associated with slightly higher noncompletion (6.5%) compared with unprovoked attacks (6.3%, p = 0.018). However, the clinical relevance of this small difference is uncertain and may simply reflect the large sample size and resulting statistical power. Noncompletion also varied significantly depending on the status of the animal (p < 0.001): it was highest (6.5%) when the animal remained alive and accessible at the end of the PEPV regimen, and lower when the animal had spontaneously died during the PEPV treatment (4.3%) or had an uncertain status between spontaneous death and slaughtered (2.5%). Among patient’s laboratory-confirmed rabid animals, noncompletion was only 2.9% (19/661), significantly lower than patients for whom the animal was not tested (p < 0.001).

Over half of exposures (54.0%, 129,423) involved the lower limbs. Most were classified as category III exposures (92.8%, 222,537), while 7.2% (17,337) were category II (Table 4). Most patients (68.1%, 163,472) initiated PEPV within one day of exposure, while 7.1% (16,926) started between 4- and 6-days post-exposure. A total of 10,198 patients received RIG, of whom 94.8% (9,672) had a category III exposure.

**Table 4:** Exposure characteristics, use of RIG, and timing of PEPV initiation.

Characteristic	Overall, N = 239,874	Complete, N = 224,525	Incomplete, N = 15,349	p-value
Wound location, n (%)				0.12
-*Head*	17,119 (7.1%)	16,080 (93.9%)	1,039 (6.1%)	
-*Lower limbs*	129,423 (54.0%)	121,164 (93.6%)	8,259 (6.4%)	
-*Upper limbs*	93,332 (38.9%)	87,281 (93.5%)	6,051 (6.5%)	
**Exposure category, n (%)**				<0.001
-*II*	17,337 (7.2%)	16,518 (95.3%)	819 (4.7%)	
-*III*	222,537 (92.8%)	208,007 (93.5%)	14,530 (6.5%)	
**Delay between exposure and first PEPV dose, n (%)**				<0.001
-*0–1 day after*	163,472 (68.1%)	153,241 (93.7%)	10,231 (6.3%)	
-*2–3 days after*	59,476 (24.8%)	55,509 (93.3%)	3,967 (6.7%)	
-*4–6 days after*	16,926 (7.1%)	15,775 (93.2%)	1,151 (6.8%)	
**Rabies Immunoglobulin (RIG), n (%)**				<0.001
-*Received*	10,198 (4.3%)	9,894 (97.0%)	304 (3.0%)	
-*Not received*	229,676 (95.7%)	214,631 (93.4%)	15,045 (6.6%)	

PEPV noncompletion did not differ significantly by wound location (p = 0.12). However, it was significantly higher among patients who initiated PEPV later than one day post-exposure (p < 0,001), had category III exposures (6.5%) compared to category II (4.7%, p < 0,001), and did not receive RIG (6.6%) compared to those who did (3.0%, p < 0,001).

### Characteristics of incomplete PEPV following exposure to laboratory-confirmed rabid animals

Among the 19 patients who did not complete their PEPV despite exposure to an animal laboratory confirmed positive for RABV, 15 (78.9%) received RIG in addition to PEPV. Three patients (15.8%) attended only a single PEPV visit, whereas 16 (84.2%) attended two visits. The number of such cases increased over time: four occurred in 2019 and 2020, five in 2021, and six in 2022 (S1 Table). Of the 19 patients, 13 were male (68.4%) and six were females (31.6%). Among males, the median age was 29 years (17.5–40 IQR) and the mean age was 32.2 years (±17.5 SD). Among the females, the median age was 22.5 years (4.5–63.5 IQR) and the mean age was 30.2 years (±26.5 SD). Only one of these patients (5.3%) visited the Battambang RPC; the remaining 18 (94.7%) received care at the Phnom Penh RPC. All exposures in this group were caused by dogs.

### Multivariate analysis of factors associated with PEPV noncompletion

Among the 17 variables tested in the bivariate analysis, 16 were significantly associated with PEPV noncompletion.

**Fig 7:**
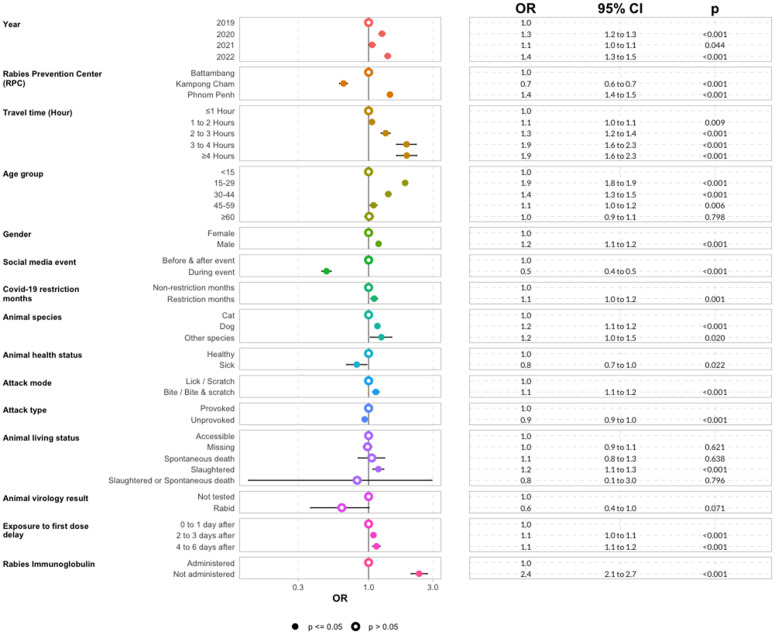
Forest plot of risk factors associated with PEPV noncompletion. Multivariate logistic regression results showing adjusted odds ratios (OR) and 95% confidence intervals (CI) for factors associated with noncompletion of PEPV. The reference category (OR=1.0) is indicated for each variable. Filled circles indicate statistically significant associations (p ≤ 0.05), while open circles represent non-significant associations (p > 0.05). Variables retained in the final model include patient demographics, exposure context, animal characteristics, health system access, and time-based contextual factors. The model was selected based on stepwise selection and lowest AIC and includes 239,874 patients with Category II and III exposures.

In the multivariate analysis, both upward and downward stepwise variable selection procedures converged on the same final model and identified 14 variables independently associated with PEPV noncompletion (Fig 7 and S2 Table). These included: year, RPC, travel time to RPC, age group, gender, social media event, Covid-19 restriction period, animal species, animal health status, attack mode, attack type, animal living status, time between exposure to first PEPV dose, and RIG administration. Exposure category was strongly associated with noncompletion in the univariate analysis (p < 0.001) but was excluded in the multivariable model during bidirectional stepwise selection. In contrast, animal laboratory results were not significantly associated with noncompletion (p = 0.066) but was included in the final model due to its contribution to improving model fit (lower AIC).

Patients seen between 2020 and 2022 were significantly more likely to be incomplete compared to those in 2019. Compared to patients attending the Battambang RPC, those at Phnom Penh RPC had higher odds of noncompletion, while patients at Kampong Cham RPC had significantly better completion. Greater travel time to an RPC was significantly associated with increased noncompletion.

Adults aged 15–59 years were more likely to be incomplete compared to children under 15 years, and males were significantly more likely to be incomplete than females. Attendance during the 2019 social media event was associated with significantly higher completion compared to other periods, whereas months with Covid-19 restrictions were associated with increased noncompletion.

Patients exposed by dogs or other animal species had higher odds of noncompletion compared to those exposed by cats. Exposure to a sick animal was associated with higher completion, while exposure to a slaughtered animal was linked to higher noncompletion. Unprovoked exposures were significantly associated with better completion, whereas bites or bites-and-scratch exposures had higher odds of noncompletion. Initiation of PEPV two or more days after exposure, as well as not receiving RIG were both significantly associated with increased odds of noncompletion.

## Discussion

We analyzed data from over 240,000 patients who received PEPV at the three IPC RPCs in Cambodia between 2019 and 2022. Overall, PEPV noncompletion remained relatively low at 6.4%, despite significant temporal and spatial variation. Multivariate analysis identified several factors significantly associated with PEPV noncompletion. These include greater travel time to the RPC, male gender, young adult age group, delayed initiation of PEPV, absence of RIG, and exposures involving a dog, a healthy animal, or a provoked attack. Noncompletion was also higher following bites or bites with scratches, when the animal was slaughtered, and during periods of Covid-19 restrictions. In contrast, PEPV completion was significantly higher during the 2019 social media event. Rates of noncompletion also varied significantly across years and RPC locations.

Findings highlighted marked differences in geographic accessibility to the three IPC RPCs. Overall, patients’ median travel times were very short (32.1 minutes), indicating that most patients visiting an RPC lived in the vicinity of the visited center, underscoring a strong impact of accessibility on RPC visits. By contrast, the theoretical median travel time for all Cambodians to the RPC that was temporally closest was 2.8 times greater (91.2 minutes). These findings are comforted by the major spatial inequities in PEPV incidence per population (Fig 5B) which show that some northern districts have PEPV attendance incidences under 10 per 100,000 inhabitants while those the closest to RPCs can reach 7,200 per 100,000 inhabitants. From a public health perspective, this indicates that most districts remain severely underserved and that more efforts need to focus on improving access to PEPV, particularly for districts located in the north-eastern half of the country.

The three centers do not play the same role, due to historical and geographical (infrastructure-related) differences. The combination of Phnom Penh’s established role as an RPC, dense transport network and central location contributes to its broad national catchment, as illustrated by its high proportion of out-of-province patients (49.0%). Opposite, Kampong Cham and Battambang only have 26.5% and 10.6% of out-of-province patients respectively, indicating a regional catchment. Furthermore, although the Kampong Cham RPC is theoretically well accessible, the median travel time for out-of-province patients was very short (39.5 minutes), indicating that its actual catchment area is much smaller than expected. Thus, the catchment of this RPC, could potentially be strengthened through greater outreach in neighboring provinces such as Kampong Thom, Kratie and Mondolkiri. Battambang RPC could also benefit, to a lesser extent, from a greater outreach in Siem Reap, Banteay Meanchey and Oddar Meanchey.

In addition to impacting RPC visits, access also impacted completion, as confirmed by the fact that PEPV noncompletion increased between patients living within 3 hours of an RPC (6.5%) and those residing more than 3 hours away (10.4%). The progressive increase in ORs across distance categories further highlight the strength and consistency of this association. These findings align with prior research identifying geographic distance or travel time as a barrier to both PEPV attendance [18,33,43–50] and PEPV completion in Cambodia and elsewhere [29,31,43,48–52].

Collectively, these findings strongly support the need for decentralization of PEPV services to reduce travel burdens and promote more equitable access to lifesaving care. Future expansion of RPCs should prioritize underserved and remote provinces with limited road connectivity, while ensuring that each new facility serves a population large enough to remain sustainable and to enhance equity in PEP access, particularly where excessive travel times may reduce treatment compliance. Baron et al (2022), showed that the opening of an RPC in Siem Reap, Banteay Meanchey, Takeo or Svay Rieng, increased the number of people living within one hour of the facility by more than 500,000 [53]. In line with this approach, efforts have been made to expand rabies prevention access in Cambodia, including the integration of a rabies PEP service into the emergency care unit of Kampot Provincial Hospital, with technical training and assistance provided by the IPC RPC. By applying to the GAVI, the Vaccine Alliance, for support in procuring rabies vaccine for PEPV, Cambodia is on the way to accomplishing another step.

Temporal trends also revealed substantial variation in PEPV noncompletion (5.0% in 2019, 7.2% in 2020, 6.0% in 2021, and 7.6% in 2022). These variations reflect several contextual events. The 2019 social media event, which followed the widely publicized death of a child from suspected rabies, was linked to a surge in RPC attendance and a two-fold reduction in noncompletion (3.2% vs. 6.8%). In contrast, the Covid-19 restriction periods in 2020–2021 were associated with decreased patient attendance and a rise in noncompletion, consistent with patterns observed globally [26,54]. Similar declines in PEPV uptake and completion during the Covid-19 pandemic have been reported in other countries [24,26,54], and have contributed to increased rabies incidence and mortality in some settings [25]. Alarmingly, the PEPV noncompletion rate in 2022 (7.6%) rose close to the 7.8% observed between 2009 and 2013, when the PEP schedule required more and visits [29]. To confirm the effect of adoption of the IPC abridged ID protocol on increasing PEPV completion, a study comparing noncompletion a few years before and after the change in regimen, considering factors shown to be associated to noncompletion, should be carried out. Furthermore, noncompletion should continue to be monitored to determine whether the upward trend persists and to identify effective strategies for reversing it.

Male patients and individuals aged 15–29 years were significantly more likely to discontinue PEPV, consistent with findings from Cambodia [29] and other settings [31,46,48,49,55]. Potential barriers in these groups may include lower health-seeking behavior, competing work or school obligations, and a tendency to underestimate the severity of rabies risk [20,29,30,32]. To improve adherence, tailored communication strategies and more flexible service delivery hours should be considered to better accommodate the needs of these higher-risk populations.

Exposure-related factors also influence PEPV noncompletion. Patients exposed to dogs were more likely to interrupt PEPV than those exposed to cats. Patients exposed to a sick animal, unprovoked attack and animals slaughtered after exposure, were more likely to complete their PEPV compared to those exposed by healthy-looking animals, provoked attack and animals which remained alive. This suggests that patients correctly identify at-risk situations aligns with existing evidence that perceived risk severity strongly influences health-seeking behavior [46,52]. Although some patients may have been aware of these risks before visiting the RPC, staff also play a role in raising awareness by informing those who have experienced particularly high-risk exposures. From a public health perspective, these findings underscore the need to emphasize completion of PEPV even following low-risk exposures.

Delayed initiation of PEPV was associated with increased dropout, potentially reflecting both lower perceived urgency and barriers to access. Patients who did not receive RIG were more likely to discontinue PEPV. While RIG is prioritized for high-risk category III exposures, its administration may also act as a proxy indicator for thorough clinical counseling, reinforcing patient adherence. Among the 661 patients exposed to laboratory-confirmed rabid animals, only 2.9% discontinued PEPV. In cases where patients were referred to IPC solely for animal testing, it is plausible that they continued PEPV at their referring facilities. However, if these patients were not referred from other health facilities, their failure to complete PEPV regimen raises concern about gaps in risk communication. Given the lethality of rabies, it is essential to ensure that patients fully understand the implications of confirmed exposure and receive appropriate counseling and follow-up. These findings highlight the urgent need for a coordinated national rabies surveillance and patient follow-up system to guarantee PEPV continuity across all levels of the health system.

Overall, Cambodia’s PEPV noncompletion rate at 6.4% remains low compared to those reported in several other countries, including Senegal, India, China and the Philippines [20,30,31,56,57]. The achievement of such a good completion among patients is likely influenced by such factors such as the adoption of the shortened three-session ID regimen [3,12,14,15,58] and implementation of partial subsidization of PEPV at IPC RPCs. Additionally, the expansion of RPCs across the country (Battambang and Kampong Cham), which improved geographic access, facilitated completion among patients living closer to these centers. The highly publicized death of a child in 2019 which was communicated by the child’s mother through Facebook, the dominant platform in Cambodia, and later amplified through Telegram, another very widely used platform, also contributed to both higher attendance and completion rates. No figures are available to quantify the number of people who viewed the post, but the influx of patients at Phnom Penh RPC was so massive that the government made an official announcement in the press to reassure the population. This event significantly raised awareness and appears to have contributed to improved adherence. Furthermore, the single payment at IPC, requiring advance payment for subsequent doses, together with the reduced vaccination fee [29], likely encouraged patients to complete the vaccination schedule. All these factors contributed to increase access to PEPV which in turn helps decrease non-completion rates. However, these benefits may be limited to IPC-managed centers and may not apply to other facilities across the country, highlighting the need for broader system-level support to sustain equitable access and adherence nationwide. Finally, another factor that may contribute to Cambodia’s high PEPV compliance rate is the proximity of patients to RPCs. It is plausible that two opposing mechanisms operate simultaneously: while RPCs are indeed located in densely populated and well-served areas, patients living farther away may either forgo PEPV or seek it from other providers.

Implementing the one-week three-session ID protocol in RPCs offers a cost-effective strategy for RPCs by enabling the treatment of approximately 33% more patients with a given stock of vials, while also reducing the time required for patients to complete their PEPV and lowering other indirect costs for vaccinees [15]. During the study period, no active surveillance was implemented to monitor outcomes in patients who did not complete PEPV. Passive outcome reporting by patients or their relatives did not reveal any cases of rabies infections among those who initiated but did not complete PEPV. IPC is planning to carry out active investigations to assess potential cases of rabies infection associated with PEPV noncompletion.

While this study provides important insights into the determinants of PEPV noncompletion, several limitations must be acknowledged. The analysis is based on data from the three IPC RPCs, which are among the largest PEPV providers in Cambodia but may not fully reflect national trends. These centers likely differ from other facilities in several key aspects: (i) they serve a larger number of patients, (ii) they use an abridged ID PEPV regimen, (iii) they are among the few centers that partially subsidize the cost of PEPV, and (iv) they operate on a one-time, upfront payment model covering all three PEPV sessions. Unfortunately, to date, no national-level PEPV data have been published in Cambodia. Establishing a comprehensive national database integrating data from all PEPV providers would be highly valuable for identifying the nationwide rabies burden with greater accuracy.

Other limitations include the absence of data on key variables such as socioeconomic status, prior rabies vaccination, and whether the exposure was investigated under an integrated bite case management (IBCM) framework. The exposure category was retrospectively reconstructed based on available information, which may have introduced classification bias. Yet, as the data were routinely collected and the questionnaires were administered by the same teams throughout the study period, any documentation or classification biases are expected to have been relatively stable over time. Nonetheless, the large number of patients included adds weight to the observations. Additionally, patients with category I exposures were not systematically recorded as they do not require PEPV, limiting our ability to assess community-level risk perception and health-seeking behavior for lower-risk incidents. While this study identified risk factors associated with PEPV noncompletion, it does not address the equally crucial question of why some exposed individuals do not seek PEPV at all. From a public health perspective, characterizing this underserved and often invisible population is essential to designing targeted awareness and outreach strategies that ensure timely and equitable access to rabies prevention services.

Finally, while the large sample size in this study reduced random error and increased the statistical power to detect very small associations, some of these associations may lack clinical or epidemiological relevance.

Despite these constraints, this study provides valuable, actionable insights into patient behaviors and system-level barriers and gaps affecting rabies PEPV completion in Cambodia. Key factors including travel time to care, age, sex, timing to PEPV initiation, and perceptions of animal health, significantly influence treatment completion. Addressing these determinants requires targeted strategies to improve completion rates and reduce preventable rabies deaths. Priority actions include raising awareness among high-risk groups, expanding geographic access to rabies prevention services, and delivering consistent, evidence-based clinical messaging. Integrating PEPV delivery into primary care and aligning with One Health surveillance systems could improve responsiveness to exposures and strengthen PEPV completion pathways. Linking human exposure data with animal health information would allow for earlier detection of high-risk exposures or outbreaks and enable more targeted interventions such as timelier and/or localized PEPV delivery, optimized vaccine distribution and public awareness campaigns. Moreover, monitoring both patient compliance and animal vaccination coverage within an integrated system would provide valuable feedback to evaluate and enhance the effectiveness of rabies control programs. Enhanced communication around the importance of PEPV completion, particularly following category III exposures or after seemingly low-risk or provoked events is essential. Future studies using qualitative or mixed-methods approaches could uncover behavioral drivers behind care-seeking and noncompletion, enabling more targeted interventions. These findings can directly inform future efforts to strengthen national rabies prevention strategies and support the development of more equitable and effective PEPV delivery systems across Cambodia.

## Conclusion

This study confirms that PEPV completion is influenced by multiple factors, with geographic access, age, and sex emerging as key determinants. In Cambodia, although overall completion rates are high, young adults, males, and individuals living far from RPCs, remain at higher risk of PEPV noncompletion. Strengthening awareness of rabies fatality risk, especially among these populations, is essential to improve treatment completion.

To achieve the ’Zero by 30’ target, scaling up a decentralized network of RPCs is critical to reduce travel-related barriers. Complementary strategies should include enhanced clinical counseling, flexible service delivery models, and integration of rabies PEPV into community-based health services. Finally, sustained progress will require a robust One Health approach. Strengthening surveillance in both human and animal populations, implementing mass dog vaccination campaigns and ensuring intersectoral coordination will be essential to achieve long-term rabies control and elimination in Cambodia.

## Supporting information

S1 File RPC patient’s questionnaire (English version). (PDF)

S1 Fig Exposure category decision tree. The figure shows the exposure categories of 239,874 patients included in the multivariate model.
(TIF)

S2 Fig Completion of patients previously immunized. The figure shows the evolution of the number of patients previously immunized from 2019 to 2022 and their PEPV completion. For previously immunized individuals ≥3 months, WHO recommends 1-site ID on days 0 and 3 or at 4-sites ID on day 0 or at 1-site IM on days 0 and 3. As IPC RPCs use an abridged ID regimen, 1-site ID on days 0 and 3 was applied. As the number of previously immunized patients increased with years, incomplete PEPV (1-visit) increased from 1.2% in 2019 to an average of 3.0% for 2020–2022. Data include all patients previously immunized ≥3 months from 2019 to 2022 (n = 17,317).
(TIF)

S3 Fig IPC RPCs travel time to districts. (A) Travel time to Battambang RPC. (B) Travel time to Kampong Cham RPC. (C) Travel time to Phnom Penh RPC. Travel time was computed using the terra and gdistance packages in R software, based on the Malaria Atlas Project, Global Motorized Friction Surface 2019. The Malaria Atlas Project maps are under the “Creative Commons Attribution 3.0 Unported License” (https://malariaatlas.org/open-access-policy/). The source of the basemap shapefile is https://gadm.org/download_country.html. The data are freely available for academic use such as publishing of academic research articles https://gadm.org/license.html.
(TIF)

S4 Fig Cambodia population map. The source of population is the 2019 national census population. The map was created using R software. The source of the basemap shapefile is https://gadm.org/download_country.html. The data are freely available for academic use such as publishing of academic research articles https://gadm.org/license.html.
(TIF)

S1 Table Patients with incomplete PEPV and exposed to a laboratory-confirmed rabid animal. (DOCX)

S2 Table Univariate and multivariate model of rabies PEPV noncompletion. (DOCX)
