# Supplementary material for: Drivers of rabies post-exposure prophylaxis noncompletion in Cambodia, 2019 to 2022
Source: PLoS Negl Trop Dis. 2025 Dec 18;19(12):e0013813. doi: 10.1371/journal.pntd.0013813 (PMC12774344; doi:10.1371/journal.pntd.0013813)
Supplement: S1 File — (PDF) [file pntd.0013813.s001.pdf]

INSTITUT PASTEUR DU CAMBODGE

RABIES PREVENTION CENTER

Sticker

Doctor ____________________________

Patient

Sex 1[_] male 2[_] female

Pregnancy 1[_] yes 2[_] no

Trimester of pregnancy

1[_] 1st 2[_] 2nd 3[_] 3rd trimester

Age ______ year(s) or ______ month(s)

Nationality 1[_] Cambo. 2[_] foreigner

Telephone N. ______________________

Email ______________________

Travelling 1[_] yes 2[_] no

Exit from Cambodia on ____________

Initial prescription:

wound care 1[_] yes 2[_] no

Anti-tetanus serum 1[_] yes 2[_] no

Tetanus vaccine 1[_] yes 2[_] no

Previously fully vaccinated against rabies

1[_] yes 2[_] no year _________

Anti-rabies serum 1[_] yes 2[_] no

Date _____________ Weights _______ Kg

Reason 1[_] lab confirmed rabid animal

2[_] suspected animal

3[_] high-risk wound

4[_] other ____________________

Rabies vaccine 1[_] yes 2[_] no

If not given, reason ___________________

D3 Animal 1[_] was killed

2[_] death of illness

3[_] disappear/lose follow-up

4[_] accessible (healthy)

D7 Animal 1[_] was killed

2[_] death of illness

3[_] disappear/lose follow-up

4[_] accessible (healthy)

Characteristics of the accident

Date _____________________

Province ______________________

Mode of exposure

1[_] bite 2[_] scratch 3[_] lick

4[_] bite & scratch

5[_] contact with human rabies case

6[_] medical staff 7[_] PrEP

8[_] special demand

Surface 1[_] intact skin 2[_] non-intact skin

3[_] mucosa

Severity 1[_] superficial 2[_] deep wound

Bleeding 1[_] yes 2[_] no

Suture 1[_] yes 2[_] no

___ stitch(s) 1[_] partial 2[_] complete

Clothing interposition 1[_] yes 2[_] no

Number of wound(s) ________________

Location of the main wounds

Loc- 1. __________________________

Loc- 2. __________________________

Loc- 3. __________________________

Characteristics of the animal and lab testing

Species 1[_] dog 2[_] cat 3[_] monkey

9[_] other ______________________

Agression 1[_] spontanous 2[_] provoke

Aspect 1[_] healthy 2[_] sick

Ownership 1[_] owned animal

2[_] stray animal

3[_] wild animal

Number of victim(s) ____________

Status of animal 1[_] was killed

2[_] death of illness

3[_] disappear/lose follow-up

4[_] accessible (healthy)

Animal tested 1[_] yes 2[_] no

Relation to sample 1[_] index 2[_] secondary

ID code of index patient __________________

(06-04-2023)
