# Supplementary figures and images for: Drivers of rabies post-exposure prophylaxis noncompletion in Cambodia, 2019 to 2022
Source: PLoS Negl Trop Dis. 2025 Dec 18;19(12):e0013813. doi: 10.1371/journal.pntd.0013813 (PMC12774344; doi:10.1371/journal.pntd.0013813)

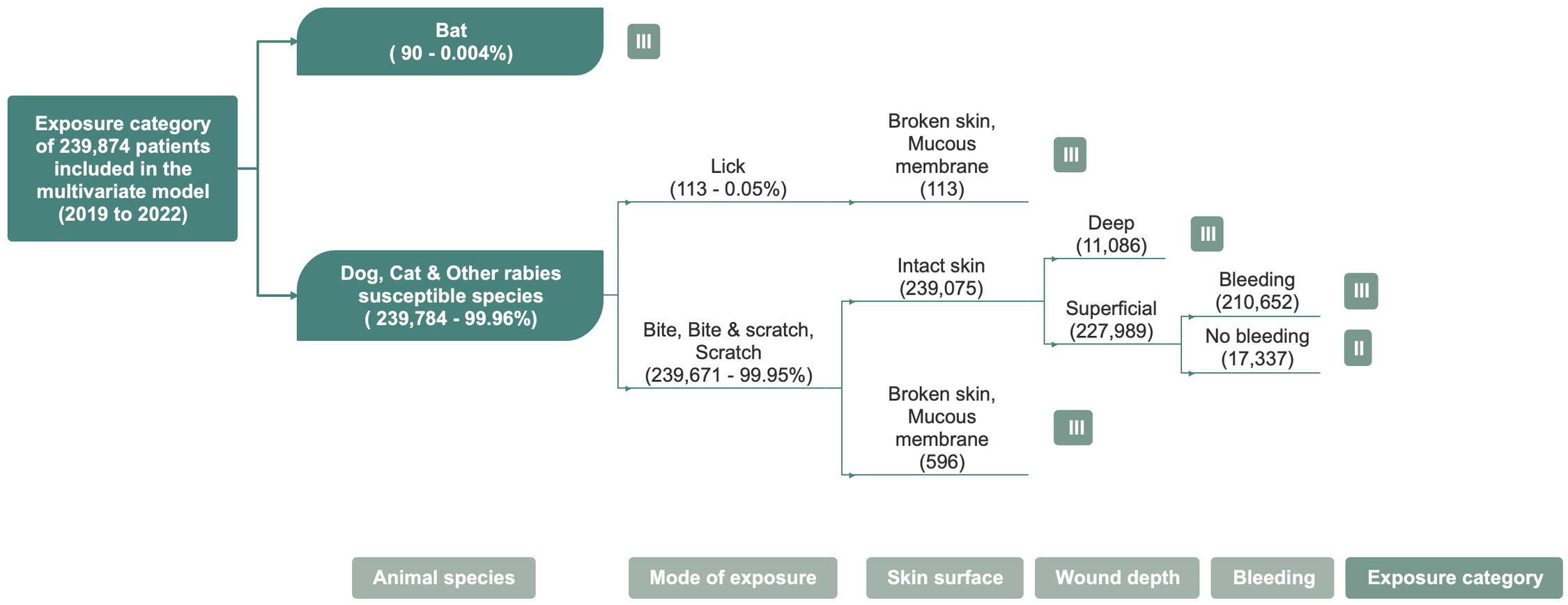

Supplement: S1 Fig — The figure shows the exposure categories of 239,874 patients included in the multivariate model. (TIF) [file pntd.0013813.s002.tif]

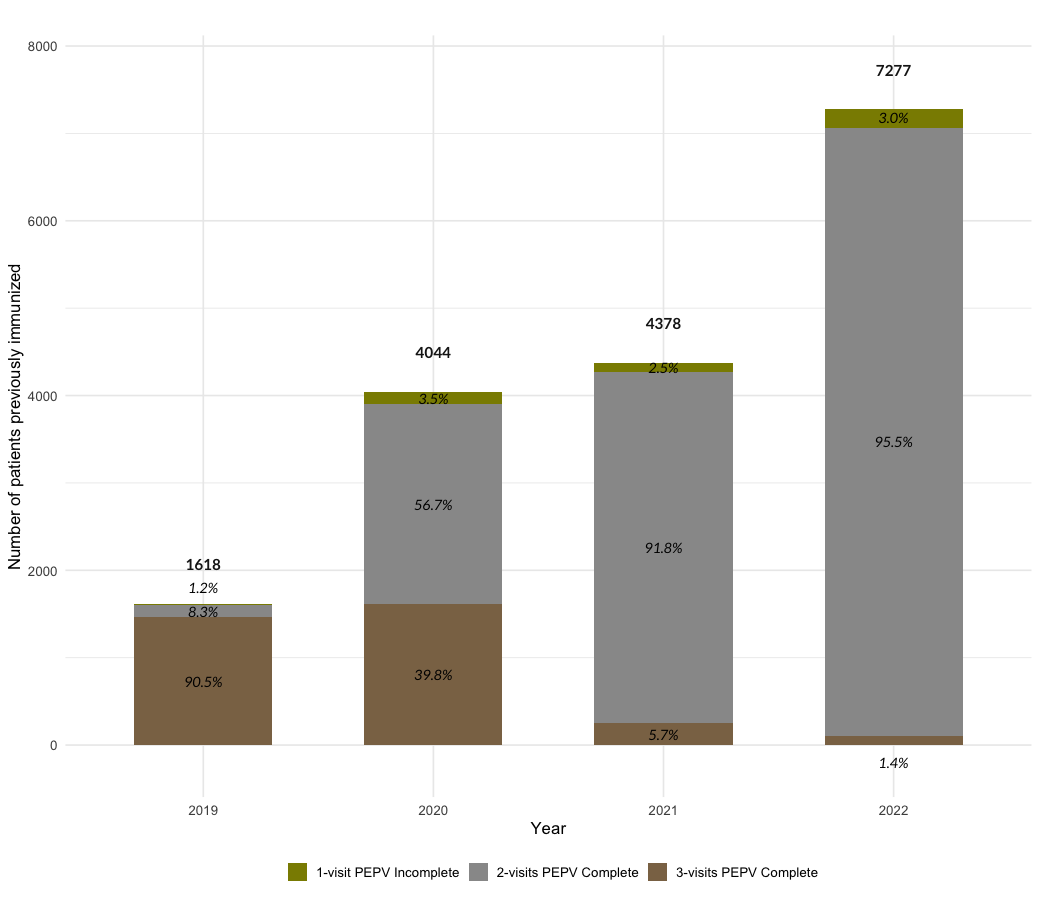

Supplement: S2 Fig — The figure shows the evolution of the number of patients previously immunized from 2019 to 2022 and their PEPV completion. For previously immunized individuals ≥3 months, WHO recommends 1-site ID on days 0 and 3 or at 4-sites ID on day 0 or at 1-site IM on days 0 and 3. As IPC RPCs use an abridged ID regimen, 1-site ID on days 0 and 3 was applied. As the number of previously immunized patients increased with years, incomplete PEPV (1-visit) increased from 1.2% in 2019 to an average of 3.0% for 2020–2022. Data include all patients previously immunized ≥3 months from 2019 to 2022 (n = 17,317). (TIF) [file pntd.0013813.s003.tif]

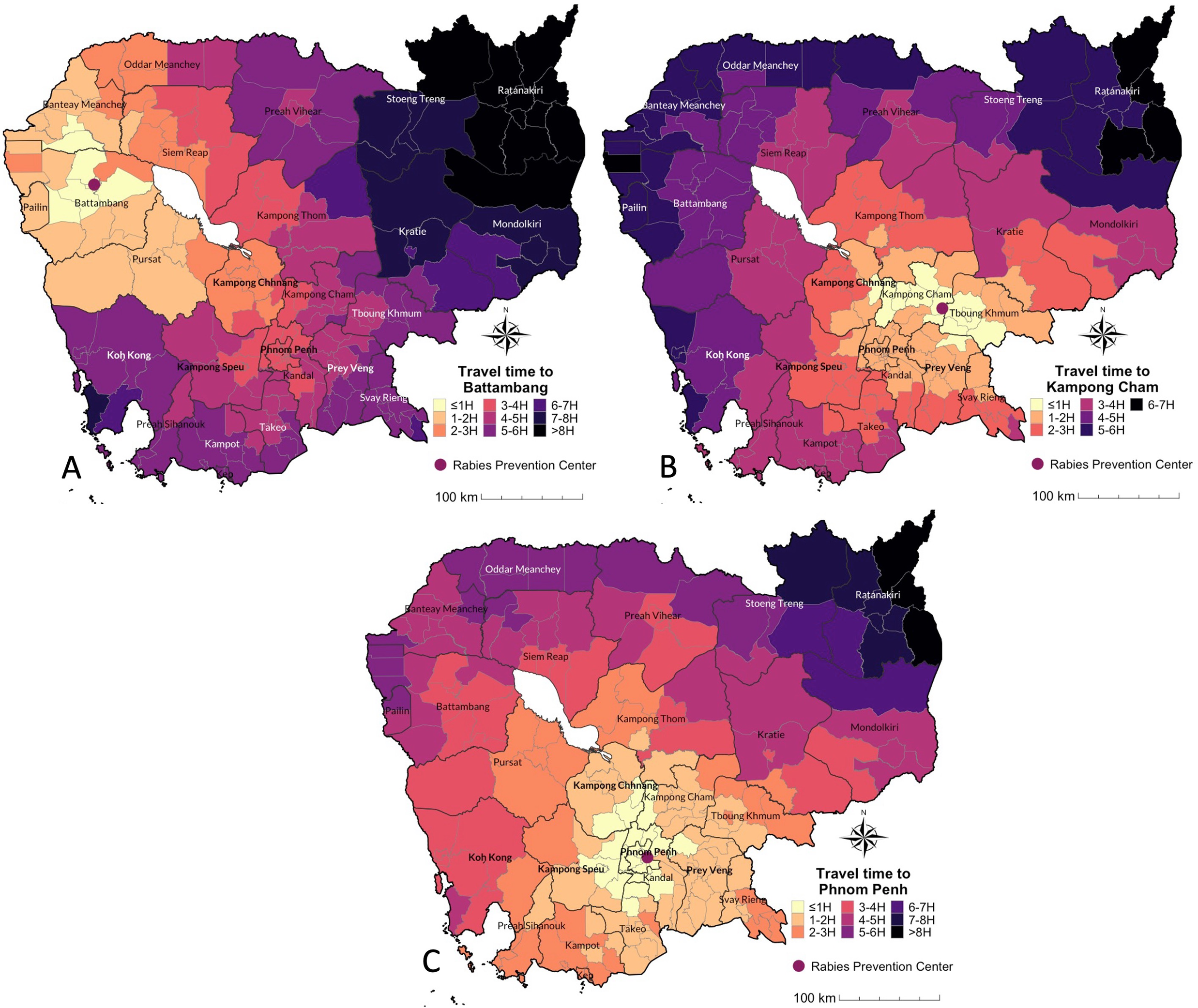

Supplement: S3 Fig — (A) Travel time to Battambang RPC. (B) Travel time to Kampong Cham RPC. (C) Travel time to Phnom Penh RPC. Travel time was computed using the terra and gdistance packages in R software, based on the Malaria Atlas Project, Global Motorized Friction Surface 2019. The Malaria Atlas Project maps are under the “Creative Commons Attribution 3.0 Unported License” (https://malariaatlas.org/open-access-policy/). The source of the basemap shapefile is https://gadm.org/download_country.html. The data are freely available for academic use such as publishing of academic research articles https://gadm.org/license.html. (TIF) [file pntd.0013813.s004.tif]

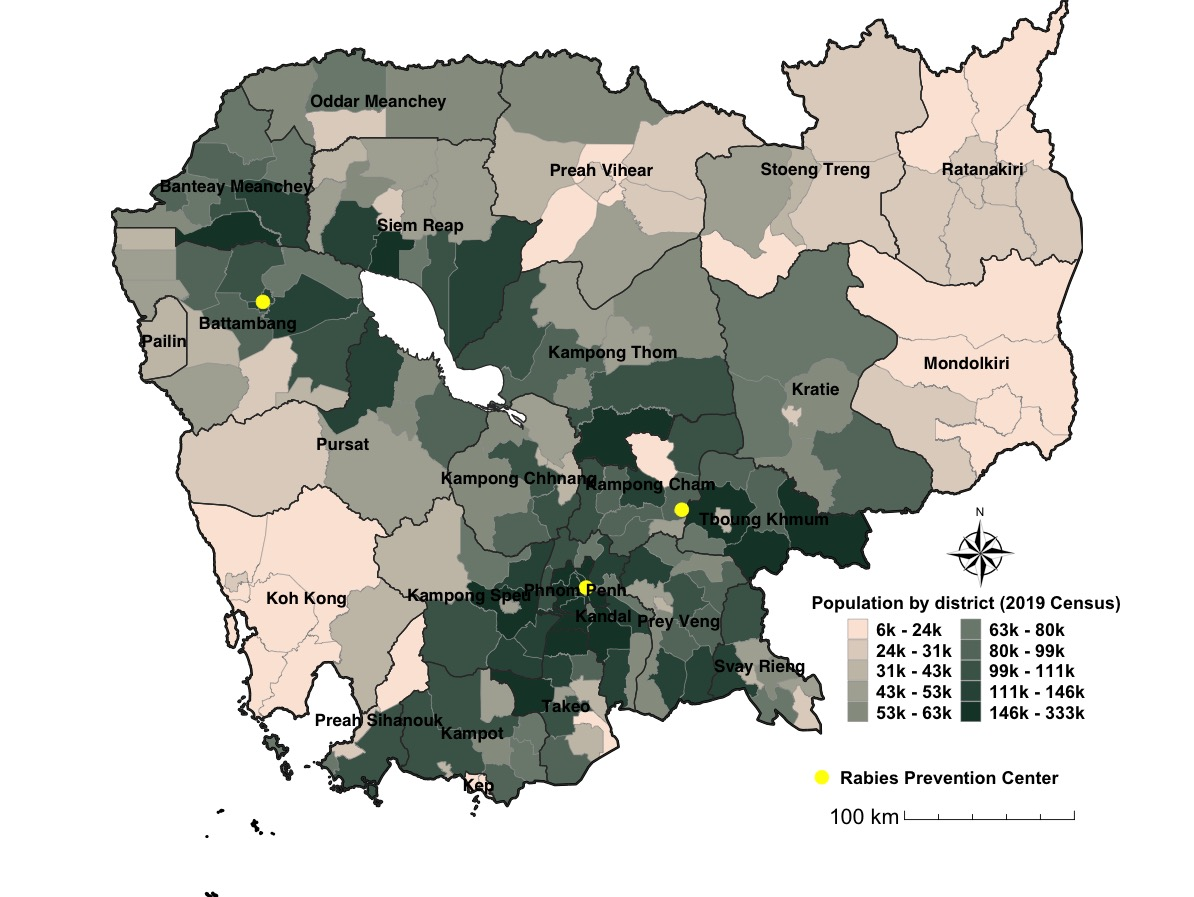

Supplement: S4 Fig — The source of population is the 2019 national census population. The map was created using R software. The source of the basemap shapefile is https://gadm.org/download_country.html. The data are freely available for academic use such as publishing of academic research articles https://gadm.org/license.html. (TIF) [file pntd.0013813.s005.tif]
