# Supplementary material for: Drivers of rabies post-exposure prophylaxis noncompletion in Cambodia, 2019 to 2022
Source: PLoS Negl Trop Dis. 2025 Dec 18;19(12):e0013813. doi: 10.1371/journal.pntd.0013813 (PMC12774344; doi:10.1371/journal.pntd.0013813)
Supplement: S1 Table — (DOCX) [file pntd.0013813.s006.docx]

**S6 Incomplete PEPV with exposure animal laboratory-confirmed positive to rabies.**

| Year | Sex | Age | Province | District | RPC | Species | Category | RIG | Delay of 1^st^ dose |
| --- | --- | --- | --- | --- | --- | --- | --- | --- | --- |
| 2019 | Male | 35 | Kampong Cham | Prey Chhor | Phnom Penh | Dog | III | Taken | 0 |
|  | Male | 29 | Kampong Speu | Kong Pisei | Phnom Penh | Dog | III | Taken | 0 |
|  | Female | 4 | Stoeng Treng | Stueng Traeng | Phnom Penh | Dog | II | Not taken | 4 |
|  | Male | 29 | Ratanakiri | Ou Ya Dav | Phnom Penh | Dog | III | Not taken | 2 |
| 2020 | Male | 16 | Takeo | Tram Kak | Phnom Penh | Dog | III | Taken | 0 |
|  | Male | 47 | Prey Veng | Me Sang | Phnom Penh | Dog | III | Taken | 1 |
|  | Male | 19 | Takeo | Samraong | Phnom Penh | Dog | III | Taken | 1 |
|  | Male | 23 | Phnom Penh | Praek Pnov | Phnom Penh | Dog | III | Taken | 1 |
| 2021 | Male | 10 | Prey Veng | Kanhchriech | Phnom Penh | Dog | III | Taken | 1 |
|  | Male | 45 | Phnom Penh | Sensok | Phnom Penh | Dog | III | Taken | 10 |
|  | Male | 32 | Kampong Thom | Baray | Phnom Penh | Dog | III | Taken | 1 |
|  | Female | 70 | Battambang | Battambang | Battambang | Dog | III | Taken | 1 |
|  | Male | 31 | Takeo | Tram Kak | Phnom Penh | Dog | III | Taken | 0 |
| 2022 | Female | 38 | Kampong Speu | Basedth | Phnom Penh | Dog | III | Taken | 2 |
|  | Female | 5 | Kampong Speu | Basedth | Phnom Penh | Dog | III | Taken | 0 |
|  | Female | 7 | Prey Veng | Svay Antor | Phnom Penh | Dog | III | Taken | 1 |
|  | Female | 57 | Svay Rieng | Romeas Haek | Phnom Penh | Dog | III | Taken | 1 |
|  | Male | 66 | Svay Rieng | Romeas Haek | Phnom Penh | Dog | III | Not taken | 2 |
|  | Male | 6 | Kratie | Sambour | Phnom Penh | Dog | III | Not taken | 1 |
