# Supplementary material for: Drivers of rabies post-exposure prophylaxis noncompletion in Cambodia, 2019 to 2022
Source: PLoS Negl Trop Dis. 2025 Dec 18;19(12):e0013813. doi: 10.1371/journal.pntd.0013813 (PMC12774344; doi:10.1371/journal.pntd.0013813)
Supplement: S2 Table — (DOCX) [file pntd.0013813.s007.docx]

|  |  | **Univariate model** | | | **Multivariate model** | | |
| --- | --- | --- | --- | --- | --- | --- | --- |
| **Characteristic** | **Incomplete PEP***^1^* | **OR** | **95% CI** | **p-value** | **OR** | **95% CI** | **p-value** |
| **Year** |  |  |  | <0.001 |  |  | <0.001 |
| 2019 | 5.0% (3,845/77,069) | — | — |  | — | — |  |
| 2020 | 7.2% (4,090/56,650) | 1.48 | 1.42, 1.55 |  | 1.26 | 1.19, 1.33 |  |
| 2021 | 6.0% (2,646/43,779) | 1.23 | 1.16, 1.29 |  | 1.06 | 1.00, 1.12 |  |
| 2022 | 7.6% (4,768/62,376) | 1.58 | 1.51, 1.65 |  | 1.39 | 1.32, 1.46 |  |
| **Rabies Prevention Center (RPC)** |  |  |  | <0.001 |  |  | <0.001 |
| Battambang | 5.5% (3,081/56,166) | — | — |  | — | — |  |
| Kampong Cham | 3.5% (1,257/36,089) | 0.62 | 0.58, 0.66 |  | 0.64 | 0.60, 0.68 |  |
| Phnom Penh | 7.5% (11,011/147,619) | 1.39 | 1.33, 1.45 |  | 1.40 | 1.34, 1.46 |  |
| **Travel time (Hour)** |  |  |  | <0.001 |  |  | <0.001 |
| ≤1 Hour | 6.3% (10,817/171,285) | — | — |  | — | — |  |
| 1 to 2 Hours | 6.0% (2,904/48,704) | 0.94 | 0.90, 0.98 |  | 1.01 | 0.96, 1.05 |  |
| 2 to 3 Hours | 7.2% (1,003/13,910) | 1.15 | 1.08, 1.23 |  | 1.19 | 1.11, 1.27 |  |
| 3 to 4 Hours | 9.5% (263/2,778) | 1.55 | 1.36, 1.76 |  | 1.67 | 1.47, 1.90 |  |
| ≥4 Hours | 11.3% (362/3,197) | 1.89 | 1.69, 2.11 |  | 1.87 | 1.67, 2.09 |  |
| **Rice harvest season** | 6.4% (8,066/126,057) |  |  | >0.9 |  |  |  |
| **Age group** |  |  |  | <0.001 |  |  | <0.001 |
| <15 | 5.2% (5,700/108,667) | — | — |  | — | — |  |
| 15-29 | 9.5% (4,159/43,765) | 1.90 | 1.82, 1.98 |  | 1.87 | 1.79, 1.95 |  |
| 30-44 | 7.3% (3,142/43,193) | 1.42 | 1.35, 1.48 |  | 1.40 | 1.34, 1.47 |  |
| 45-59 | 5.5% (1,499/27,305) | 1.05 | 0.99, 1.11 |  | 1.09 | 1.02, 1.15 |  |
| ≥60 | 5.0% (849/16,944) | 0.95 | 0.88, 1.03 |  | 1.01 | 0.94, 1.09 |  |
| **Gender** |  |  |  | <0.001 |  |  | <0.001 |
| Female | 5.9% (7,256/123,290) | — | — |  | — | — |  |
| Male | 6.9% (8,093/116,584) | 1.19 | 1.15, 1.23 |  | 1.19 | 1.15, 1.23 |  |
| **Social media event** |  |  |  | <0.001 |  |  | <0.001 |
| Before & after event | 6.8% (14,583/215,643) | — | — |  | — | — |  |
| During event | 3.2% (766/24,231) | 0.45 | 0.42, 0.48 |  | 0.48 | 0.45, 0.53 |  |
| **Covid-19 restriction months** |  |  |  | <0.001 |  |  | 0.001 |
| Non-restriction months | 6.3% (13,569/215,574) | — | — |  | — | — |  |
| Restriction months | 7.3% (1,780/24,300) | 1.18 | 1.12, 1.24 |  | 1.10 | 1.04, 1.17 |  |
| **Animal species** |  |  |  | <0.001 |  |  | <0.001 |
| Cat | 6.0% (5,751/95,760) | — | — |  | — | — |  |
| Dog | 6.7% (9,470/142,093) | 1.12 | 1.08, 1.16 |  | 1.16 | 1.12, 1.21 |  |
| Other species | 6.3% (128/2,021) | 1.06 | 0.88, 1.26 |  | 1.24 | 1.03, 1.49 |  |
| **Animal health status** |  |  |  | <0.001 |  |  | 0.019 |
| Healthy | 6.5% (15,146/234,126) | — | — |  | — | — |  |
| Sick | 3.5% (203/5,748) | 0.53 | 0.46, 0.61 |  | 0.82 | 0.68, 0.97 |  |
| **Exposure mode** |  |  |  | <0.001 |  |  | <0.001 |
| Lick / Scratch | 5.6% (1,611/28,571) | — | — |  | — | — |  |
| Bite / Bite & scratch | 6.5% (13,738/211,303) | 1.16 | 1.10, 1.23 |  | 1.13 | 1.07, 1.20 |  |
| **Attack type** |  |  |  | 0.018 |  |  | <0.001 |
| Provoked | 6.5% (7,332/112,374) | — | — |  | — | — |  |
| Unprovoked | 6.3% (8,017/127,500) | 0.96 | 0.93, 0.99 |  | 0.94 | 0.90, 0.97 |  |
| **Animal living status** |  |  |  | <0.001 |  |  | 0.020 |
| Accessible | 6.5% (13,773/210,521) | — | — |  | — | — |  |
| Missing | 5.2% (929/17,943) | 0.78 | 0.73, 0.83 |  | 0.98 | 0.91, 1.05 |  |
| Spontaneous death | 4.3% (89/2,054) | 0.65 | 0.52, 0.80 |  | 1.05 | 0.83, 1.31 |  |
| Slaughtered | 6.0% (556/9,275) | 0.91 | 0.83, 0.99 |  | 1.17 | 1.07, 1.29 |  |
| Slaughtered or Spontaneous death | 2.5% (2/81) | 0.36 | 0.06, 1.15 |  | 0.82 | 0.13, 2.94 |  |
| **Animal virology result** |  |  |  | <0.001 |  |  | 0.066 |
| Not tested | 6.4% (15,330/239,213) | — | — |  | — | — |  |
| Rabid | 2.9% (19/661) | 0.43 | 0.26, 0.66 |  | 0.64 | 0.38, 1.03 |  |
| **Exposure category** |  |  |  | <0.001 |  |  |  |
| II | 4.7% (819/17,337) | — | — |  |  |  |  |
| III | 6.5% (14,530/222,537) | 1.41 | 1.31, 1.52 |  |  |  |  |
| **Exposure to first dose delay** |  |  |  | <0.001 |  |  | <0.001 |
| 0 to 1 day after | 6.3% (10,231/163,472) | — | — |  | — | — |  |
| 2 to 3 days after | 6.7% (3,967/59,476) | 1.07 | 1.03, 1.11 |  | 1.08 | 1.04, 1.13 |  |
| 4 to 6 days after | 6.8% (1,151/16,926) | 1.09 | 1.03, 1.16 |  | 1.14 | 1.07, 1.21 |  |
| **Rabies Immunoglobulin** |  |  |  | <0.001 |  |  | <0.001 |
| Administered | 3.0% (304/10,198) | — | — |  | — | — |  |
| Not administered | 6.6% (15,045/229,676) | 2.28 | 2.04, 2.57 |  | 2.39 | 2.08, 2.75 |  |
| No |  | — | — |  |  |  |  |
| Yes |  | 1.00 | 0.97, 1.03 |  |  |  |  |
| *^1^*% (n/N); Median (Q1, Q3) | | | | | | | |
| Abbreviations: CI = Confidence Interval, OR = Odds Ratio | | | | | | | |
